# Exercise capacity of vegan, lacto-ovo-vegetarian and omnivorous recreational runners

**DOI:** 10.1186/s12970-019-0289-4

**Published:** 2019-05-20

**Authors:** Josefine Nebl, Sven Haufe, Julian Eigendorf, Paulina Wasserfurth, Uwe Tegtbur, Andreas Hahn

**Affiliations:** 1 Faculty of Natural Sciences, Institute of Food Science and Human Nutrition, Leibniz University Hannover, 30159 Hannover, Germany; 2 Institute of Sports Medicine, Hannover Medical School , 30625 Hannover, Germany

**Keywords:** Recreational runners, Vegan, Vegetarian, Plant-based diets, Exercise capacity

## Abstract

**Background:**

In search of the right nutrition for the athlete, numerous nutritional strategies and diets were discussed over time. However, the influence of plant-based diets, especially veganism, on exercise capacity has not been clarified.

**Methods:**

We conducted a cross-sectional study to compare the exercise capacity of vegan (VEG, *n* = 24), lacto-ovo-vegetarian (LOV, *n* = 26) and omnivorous (OMN, *n* = 26) recreational runners. To determine maximal exercise capacity, participants performed an incremental exercise test on a bicycle ergometer until voluntary exhaustion. During the test capillary blood samples were taken at several time points for the measurement of arterial lactate [lac] and glucose [glc] concentrations. To determine nutrient intake, a 24 h dietary recall was conducted.

**Results:**

The groups showed comparable training habits in terms of training frequency (mean 3.08 ± 0.90 time/wk., *p* = 0.735), time (mean 2.93 ± 1.34 h/wk., *p* = 0.079) and running distance (mean 29.5 ± 14.3 km/wk., *p* = 0.054). Moreover, similar maximum power output (P_maxBW_) was observed in all three groups (OMN: 4.15 ± 0.48 W/kg, LOV: 4.20 ± 0.47 W/kg, VEG: 4.16 ± 0.55 W/kg; *p* = 0.917) and no differences regarding [lac] throughout the exercise test and maximum lactate could be observed between the groups (OMN: 11.3 ± 2.19 mmol/l, LOV: 11.0 ± 2.59 mmol/l, VEG: 11.9 ± 1.98 mmol/l; *p* = 0.648).

**Conclusion:**

The data indicate that each examined diet has neither advantages nor disadvantages with regard to exercise capacity. These results suggest that a vegan diet can be a suitable alternative for ambitious recreational runners.

**Trial registration:**

German Clinical Trials Register (DRKS00012377). Registered on 28 April 2017

## Background

Most endurance athletes are interested in diets that positively affect exercise capacity and health, reduce body fat and promote the development of lean muscle mass [1]. Already thousands of years ago, the diet of athletes was seen as an important mean to increase performance [2]. While in the past meat was seen as an irreplaceable performance-enhancing food [3], today the trend is developing in the opposite direction: From partial exclusion (lacto−/ovo−/lacto-ovo-vegetarians) to the total elimination (veganism) of animal products from the diet. Since the prevalence of ambitious runners following plant-based diets is increasing [4, 5],the impact of those diets with regard to athletes performance and health is becoming of growing interest [6].

Due to the favorable impact on health [7–12] it could be assumed that performance parameters are also influenced by plant-based diets based on a broad variety of foods. Parameters to analyze exercise capacity include maximum power output and lactate concentration, with the latter particularly important with increasing exercise intensities and the associated increased in lactate production by anaerobic energy supply. Since vegetarian diets are characterized by higher intake of carbohydrates one could hypothesize that there are favorable effects on exercise capacity [13–16]. Also, the increased intake of antioxidants in plant-based diets might have positive effects on exercise-induced oxidative stress [15, 16]. On the other hand, it has been shown that vegetarians and especially vegans have lower ferritin levels whereas hemoglobin levels and the prevalence of iron deficiency anemia are generally indistinguishable from omnivores [15, 17, 18]. In addition, especially a vegan diet is usually characterized by low intake of protein, creatine and carnitine, which could negatively impact performance [19, 20].

To date, the impact of a plant-based diet on athletic performance is not clearly understood. Recent case reports showed that even vegan athletes can reach top athletic performances [21, 22]. Other studies dealing with a vegetarian and vegan diet related to sport are questionnaire-based and do not include nutritional or sports medical diagnostics [4, 5, 23, 24]. Further studies assessing nutritional and sports medical parameters are outdated [25] or did not differentiate between vegetarians and vegans [14]. A recent cross-sectional study described the oxidative status of male vegan, vegetarian and omnivorous recreational athletes but did not examine exercise capacity [26]. In addition to cross-sectional studies, there are also a few intervention studies that examine the effect of a vegetarian diet on athletic performance. However, their impact is low due to the low number of subjects and short intervention periods [27–31].

As a consequence, we conducted a study to test the hypothesis that there are no differences in exercise performance of omnivorous, lacto-ovo-vegetarian and vegan recreational runners.

## Subjects and methods

### Participants

Seventy-six healthy omnivorous (OMN, *n* = 26), lacto-ovo-vegetarian (LOV, *n* = 24) and vegan (VEG, *n* = 24) recreational runners between 18 and 35 years conducted laboratory physical exercise tests (for details see Table 1).

**Table 1:** Characterization of the study population

	OMN (*n* = 26)	*P* value OMN-LOV	LOV (n = 26)	*P* value LOV-VEG	VEG (*n* = 24)	*P* value OMN-VEG	*P* value 3 groups
Age, years	27.2 ± 4.05	–	27.6 ± 4.31	–	27.5 ± 4.26	–	0.937^a^
Sex	m = 10, w = 16	–	m = 10, w = 16	–	m = 9, w = 15	–	0.997^c^
BMI, kg/m^2^	22.2 ± 1.73	–	21.6 ± 1.98	–	22.0 ± 2.23	–	0.559^a^
Duration of diet							0.001^d^
< 0.5 years (%)	0		0		0		
0.5–1 year (%)	0		15.4		20.8		
1–2 years (%)	3.8		11.5		12.5		
2–3 years (%)	0		7.7		29.2		
> 3 years (%)	96.2		65.4		37.5		
Training habits							
Training frequency per week	3.04 ± 0.98	–	3.19 ± 0.90	–	3.00 ± 0.85	–	0.735^a^
Running distance per week, km	28.03 ± 14.66	–	34.41 ± 14.53	–	25.53 ± 12.30	–	0.054^a^
Running time per week, h	2.72 ± 1.11	–	3.38 ± 1.43	–	2.65 ± 1.38	–	0.079^a^
Heart rate during training, bpm	159.91 ± 8.89	–	151.99 ± 12.29	–	156.46 ± 12.52	–	0.173^b^
Body composition							
TBW, L	39.3 ± 6.74	–	38.5 ± 6.40	–	38.9 ± 8.20	–	0.864^a^
LBM, kg	53.7 ± 9.21	–	52.6 ± 8.75	–	53.2 ± 11.2	–	0.866^a^
Body fat, %	21.5 ± 5.91	–	21.8 ± 6.19	–	20.7 ± 5.79	–	0.797^b^
BCM, %	54.5 ± 3.33	0.043^c^	52.3 ± 3.25	n.s.	52.5 ± 2.76	n.s.	0.029^b^

*OMN* = omnivorous athletes, *LOV* = lacto-ovo-vegetarian athletes, *VEG* = vegan athletes, *n.s.* = not significant, *TBW* = total body water, *LBM* = lean body mass, *BCM* = body cell mass. Data are presented as mean (SD)

^a^Kruskal Wallis test

^b^One-way ANOVA

^c^Post hoc test

^d^Chi square test

Subjects were recruited from the general population in Hannover, Germany, via local running events, online running communities as well as online vegetarian and vegan communities. To avoid seasonal influences, the recruitment happened batchwise from May until December 2017. Participants were matched according to age and gender.

Participants were categorized upon enrolling for the study. To categorize subjects as omnivorous, lacto-ovo-vegetarian and vegan, a questionnaire which included questions about their current diet had to be completed. Additionally, usually consumed food groups were queried, to avoid subjectively wrong classifications. Subjects were “omnivorous”, if they consumed cereals, plant-based foods, legumes, milk and dairy products, eggs, as well as fish, meat and meat products. “Lacto-ovo-vegetarians” were defined as they consumed cereals, plant-based foods, legumes, milk and dairy products, and eggs. “Vegans” were characterized by consumption of cereals, plant-based foods, and legumes.

Subjects were selected based on the following inclusion criteria: omnivorous, lacto-ovo-vegetarian or vegan diet for at least half a year, body mass index (BMI) between 18.5 and 25.0 kg/m^2^ and regular run training 2 to 5 times per week. Training duration, distance and time of a typical exercise training week were documented via self-reporting data. The following criteria led to exclusion: any cardiovascular, metabolic or malignant disease, diseases regarding the gastrointestinal tract, pregnancy, nutrient intolerances as well as addiction to drugs or alcohol. The use of dietary supplements in physiological doses did not lead to exclusion, except performance-enhancing substances (e.g. creatine).

Ethical approval was provided by the Ethics Committee at the Medical Chamber of Lower Saxony (Hannover, Germany). The study was conducted in accordance with the Declaration of Helsinki. All subjects gave their written informed consent. This study is registered in the German Clinical Trial Register (DRKS00012377).

### Study procedure

First of all, the measurement of body weight (seca®, Hamburg, Germany) was carried out lightly clothed and without shoes. Second, an electrocardiogram in rest and a short medical examination were carried out and evaluated by an experienced cardiologist to make sure that the participants could join the exhaustion test. After the medical examination, a 24 h dietary recall was conducted by qualified personnel before the exercise test started. To analyze the nutrient and energy intake of the 24 h recall, the nutrition organization software PRODI® (Nutri-Science GmbH, Freiburg, Germany) was used.

The primary outcome maximum exercise capacity was measured as maximum power related to body weight (P_maxBW_) reached in the graded exercise test (GXT). Secondary outcomes included maximum power output related to lean body mass (P_maxLBM_), maximal and submaximal lactate [lac] and glucose [glc] concentrations during the GXT. The GXT was performed until voluntary exhaustion on a bicycle ergometer (Excalibur, Lode B.V., Groningen, Netherlands). Prior to physical performance test, participants were asked not to do any strenuous activities 24 h prior the performance diagnostics. Subjects were requested to maintain their usual diet. After a warm-up period of 6 min at 50 W, the workload increased by 16.7 W per minute. Heart rate (HR) was measured continuously beat-to-beat throughout all testing sessions with an HR-monitor (RS800 CX Polar, Finland). To ensure that the subjects achieve their maximum performance, they were verbally motivated by personnel, but they were not allowed to get out of the saddle. During the test, arterialized capillary blood samples were taken from the earlobe at rest, every 50 W and at termination of the test. Samples were immediately transferred into a glucose/lactate hemolysis solution (EKF-diagnostics GmbH, Barleben, Germany). Lactate and glucose concentrations were directly analyzed by a lactate/glucose biosensor (Biosen S-Line Lab+, EKF-diagnostics GmbH, Barleben, Germany).

On a separate day (at least 48 h apart), lean body mass (to a nearest of 100 g), total body water, body cell mass and relative body fat (%) were measured using a bipolar bioelectrical impedance analyzer (BIA) (Nutriguard M, Data Input Company, Darmstadt, Germany) as well as the relative software NutriPlus© 5.4.1 (Data Input Company, Darmstadt, Germany). BIA measurements were carried out in a fasting state. The participants were in lying position for 5 min before the measurement to ensure a uniform distribution of body fluids. In order to guarantee an accurate measurement, the subjects were instructed previously to lie relaxed and steady during the measurement and slightly bend their limbs from the torso. The measurement was carried out by a professional nutritionist.

### Data analysis and statistical methods

Statistical analyses were performed using SPSS software (IBM SPSS Statistics 24.0; Chicago, IL, USA). Results are shown in mean ± standard deviation (SD). First, normal distribution was checked by using the Kolmogorov-Smirnov test. If data were normally distributed, one-way analysis of variance (ANOVA) was used to evaluate differences between the three diet groups. Further, to analyze data with non-normally distribution, Kruskal Wallis test was performed. Additionally, if there were significant differences between the groups, post hoc test with Bonferroni correction was conducted. Moreover, the chi-square test was used to compare differences between the frequency distribution of the three groups. Associations between parametric data were computed via Pearson, non-parametric data via Spearman’s rho correlation. *P* values ≤0.05 were set as statistically significant.

## Results

From a total of 76 runners 26 were included in the OMN, 26 in the LOV and 24 in the VEG group. Men and women were equally distributed (*p* = 0.997, Table 1). Mean age (27.4 ± 4.16 y) and BMI (21.9 ± 1.97 kg/m^2^) did not differ significantly between the groups. Additionally, all three groups did not differ in their training frequency, running time and running distance (Table 1). Moreover, none of the subjects consumed tobacco on a regular basis.

### Exercise capacity

**Fig. 1:**
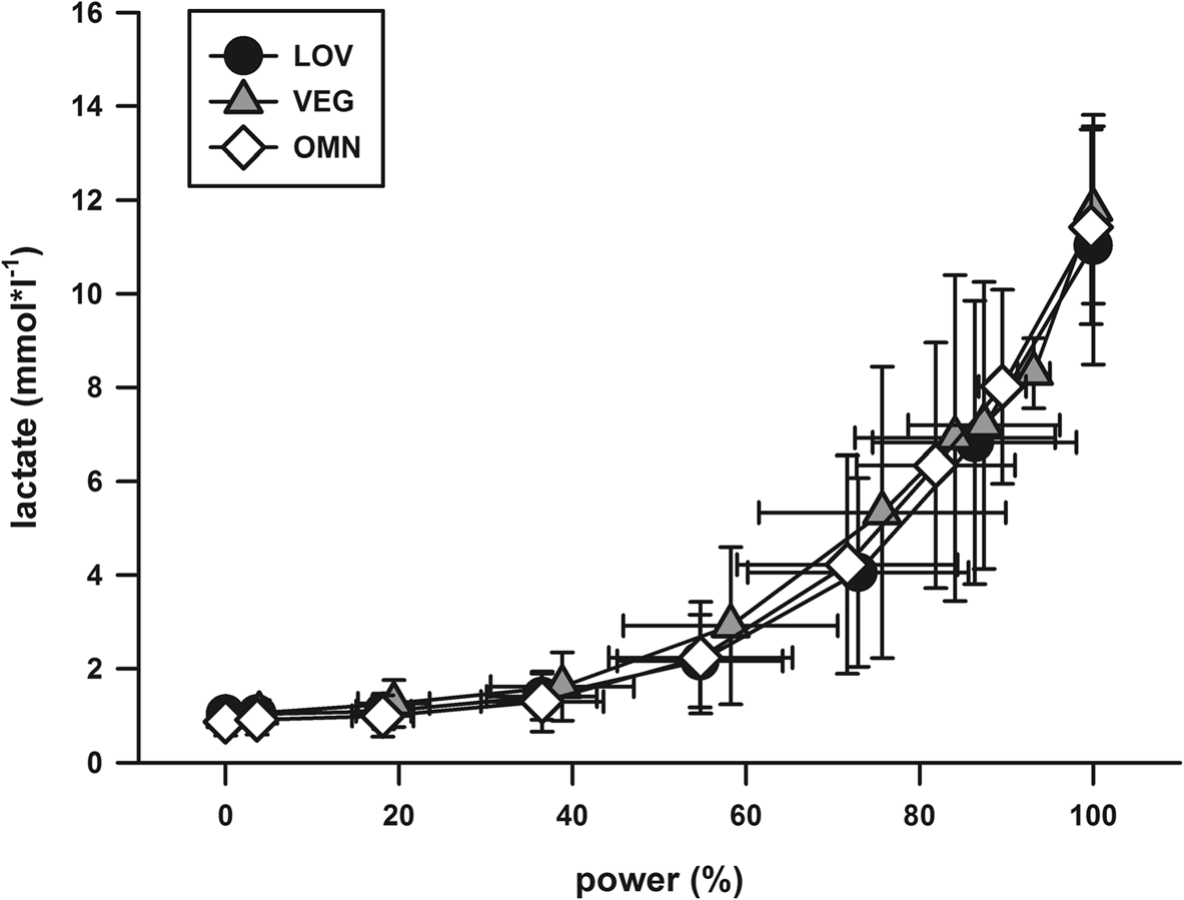
Lactate concentrations in relation to relative power output. No differences were found between the groups in either the submaximal or the maximal [lac] values. OMN = omnivorous athletes, LOV = lacto-ovo-vegetarian athletes, VEG = vegan athletes. Data are presented as mean (SD)

**Fig. 2:**
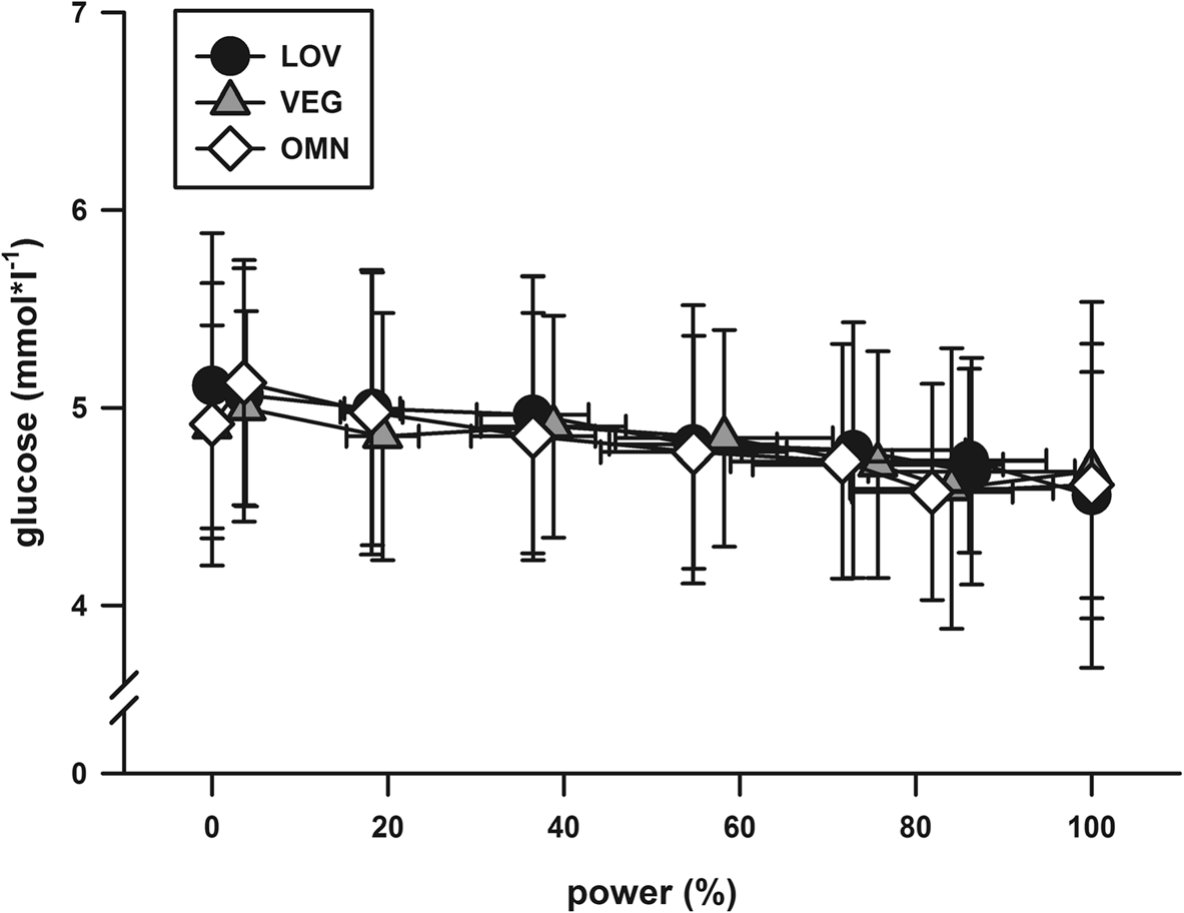
Glucose concentrations in relation to relative power output. No differences were found between the groups in [glu] values. OMN = omnivorous athletes, LOV = lacto-ovo-vegetarian athletes, VEG = vegan athletes. Data are presented as mean (SD)

For P_maxBW_ (OMN: 4.15 ± 0.48, LOV: 4.20 ± 0.47, VEG: 4.16 ± 0.55 W/kg BW) and P_maxLBM_ (OMN: 5.29 ± 0.48, LOV: 5.39 ± 0.52, VEG: 5.26 ± 0.58 W/kg LBM), there were no significant differences between the groups (*p* = 0.917 and *p* = 0.696 for P_maxBW_ and P_maxLBM_, respectively). When comparing total men and women, men showed higher P_maxBW_ (4.41 ± 0.45 W/kg vs. 4.02 ± 0.47 W/kg, *p* = 0.001). Additionally, there were no differences between performance-related parameters when comparing only women (P_maxBW_ women: OMN: 3.99 ± 0.46, LOV: 4.06 ± 0.44, VEG: 4.02 ± 0.53 W/kg, *p* = 0.910) or men (P_maxBW_ men: OMN: 4.41 ± 0.41 W/kg, LOV: 4.43 ± 0.46, VEG: 4.39 ± 0.52, *p* = 0.979) between the three study groups. Training frequency, running time and distance were not associated with P_maxBW_ in any group. In all three groups, training frequency, running time and distance were significantly correlated. Both, the maximum (*p* = 0.648) and the submaximal [lac] revealed no differences between the groups (Fig. 1). Similarly, we found no differences in maximum (*p* = 0.960) and submaximal [glc] (Fig. 2).

### Dietary intake

The 24 h dietary recall revealed some differences in nutrient intake between the groups (Table 2). While total energy and protein intake were comparable in all three groups, VEG consumed significantly higher amounts of carbohydrates, fiber, magnesium, iron, folate and vitamin E compared to OMN and also LOV. However, consumption of dietary fat and vitamin B_12_ was significantly lower in VEG compared to the two other groups.

**Table 2:** Nutrient intake of the study population determined via 24 h dietary recall

	OMN (*n* = 26)	*P* value OMN-LOV	LOV (*n* = 26)	*P* value LOV-VEG	VEG (*n* = 24)	*P* value OMN-VEG	*P* value 3 groups	Reference values* (m/f)
Energy intake, MJ	9.49 ± 3.52	–	9.04 ± 3.73	–	9.17 ± 3.53	–	0.898^a^	
Macronutrients								
Carbohydrate, EN%	49.4 ± 10.7	n.s.	48.7 ± 9.96	0.008^c^	58.9 ± 14.3	0.016^c^	0.004^a^	50–55
Carbohydrate, g/kg BW	3.87 ± 1.34	–	3.76 ± 1.55	–	4.66 ± 1.79	–	0.095^a^	
Protein, EN%	17.0 ± 6.13	–	16.5 ± 7.42	–	13.9 ± 3.97	–	0.202^b^	12–15
Protein, g/kg BW	1.37 ± 0.65	–	1.29 ± 0.81	–	1.10 ± 0.57	–	0.252^b^	0.8
Fat, EN%	32.2 ± 11.1	n.s.	32.7 ± 9.63	0.026^c^	24.8 ± 10.6	0.043^c^	0.015^a^	30–35
Fiber, g	29.6 ± 15.0	n.s.	31.6 ± 12.9	< 0.001^c^	52.1 ± 23.6	< 0.001^c^	< 0.001^a^	≥ 30
Minerals								
Sodium, g	2.85 ± 1.89	n.s.	2.23 ± 1.23	0.036^c^	1.40 ± 1.00	0.003^c^	0.003^b^	1.5
Potassium, g	3.03 ± 1.14	n.s.	3.07 ± 1.14	n.s.	4.38 ± 2.08	0.041^c^	0.031^b^	4^d^
Calcium, mg	1102 ± 619	n.s.	1252 ± 546	0.035^c^	903 ± 554	n.s.	0.042^b^	1000
Magnesium, mg	429 ± 144	n.s.	443 ± 161	0.014^c^	639 ± 294	0.008^c^	0.004^b^	350/300
Iron, mg	15.3 ± 11.9	n.s.	12.7 ± 5.35	0.029^c^	18.4 ± 7.86	n.s.	0.018^b^	10/15
Zinc, mg	12.0 ± 6.16	–	10.1 ± 3.93	–	10.4 ± 4.99	–	0.752^b^	10/7
Phosphorus, mg	1444 ± 674	–	1458 ± 685	–	1341 ± 634	–	0.871^b^	700
Copper, mg	2.12 ± 1.55	n.s.	2.15 ± 0.81	n.s.	2.90 ± 1.25	0.002^c^	0.002^b^	1.0–1.5
Vitamins								
Thiamine, mg	1.42 ± 0.80	n.s.	1.39 ± 1.22	0.036^c^	1.82 ± 0.85	n.s.	0.037^b^	1.2/1.0
Riboflavin, mg	1.58 ± 1.17	–	1.80 ± 1.58	–	1.23 ± 0.66	–	0.346^b^	1.4/1.1
Niacin, mg	35.4 ± 23.5	–	30.7 ± 20.1	–	31.1 ± 14.1	–	0.677^b^	15/12
Pyridoxine, mg	2.00 ± 1.84	n.s.	1.71 ± 1.62	0.034^c^	2.32 ± 1.23	n.s.	0.033^b^	1.5/1.2
Cobalamin, μg	5.05 ± 5.44	n.s.	3.61 ± 3.12	< 0.001^c^	0.76 ± 0.34	< 0.001^c^	< 0.001^b^	4
Biotin, μg	53.7 ± 39.6	n.s.	63.2 ± 44.5	n.s.	72.5 ± 31.4	0.017^c^	0.021^b^	30-60^d^
Pantothenic acid, mg	5.73 ± 5.21	–	6.15 ± 6.13	–	5.92 ± 3.23	–	0.298^b^	6^d^
Folate, μg	303 ± 196	n.s.	346 ± 244	0.025^c^	452 ± 177	0.002^c^	0.002^b^	300
Retinol equivalents, mg	1.41 ± 1.53	–	1.74 ± 1.50	–	2.21 ± 2.68	–	0.314^b^	1.0/0.8
Ascorbic acid, mg	140 ± 151	n.s.	148 ± 142	n.s.	237 ± 165	0.024^c^	0.018^b^	110/95
Vitamin D, μg	1.97 ± 3.30	–	2.07 ± 1.87	–	1.32 ± 1.84	–	0.129^b^	20
Vitamin E, mg	11.7 ± 6.44	n.s.	13.3 ± 10.8	0.032^c^	21.1 ± 13.6	0.018^c^	0.009^b^	14/12^d^

*OMN* = omnivorous athletes, *LOV* = lacto-ovo-vegetarian athletes, *VEG* = vegan athletes, *MJ* = mega joule, *BW* = body weight, *n.s.* = not significant

*Reference values of the German, Austrian and Swiss Nutrition Societies [39]. Nutrient intake excluding supplement intake. Data are presented as mean (SD)

^a^One-way ANOVA

^b^Kruskal Wallis test

^c^Post hoc test

^d^Estimated values

## Discussion

To the best of our knowledge, this was the first investigation providing a differential analysis of exercise capacity and lactate/glucose concentrations of vegan, lacto-ovo-vegetarian and omnivorous recreational runners. Our findings that VEG, OMN and LOV show no significant differences in maximum exercise capacity as measured by P_maxBW_ indicate that the evaluated diets do not have detrimental effects on exercise performance in recreational runners. In this regard the evaluation of the 24 h dietary recalls showed a sufficient supply in most nutrients.

Previous studies focused on the comparison between vegetarian and omnivorous athletes and observed no differences regarding physical performance [32]. An earlier study examining vegetarians and omnivores, who underwent a cycle ergometer stress test to determine the aerobic capacity and a Wingate test to estimate anaerobic capacity found no differences in performance parameters [25]. A recent study testing the physical performance of 35 vegetarian and 35 omnivorous endurance athletes, observed a 13% greater maximal oxygen consumption (VO_2 max_) in female vegetarians than in omnivores, while no differences were found in males [14]. Our study cannot directly be compared as Lynch and colleagues examined VO_2max_ and performed their exercise tests on a treadmill. Notably, previous studies did not focus on vegans and no lactate/glucose measurements as markers of anaerobic metabolism were carried out. Our study extends existing knowledge as we could show that vegan and vegetarian runners did not differ from omnivores in terms of exercise capacity and glucose utilization from low to maximum effort.

Few studies investigating the effect of a short-term lacto-ovo-vegetarian diet on performance revealed different results. A 6 and 5 weeks defined lacto-ovo-vegetarian diet did not have a significant influence on aerobic capacity or repeated sprint ability, respectively, compared to controls [27, 33]. In contrast, Hietavala et al. examined the effect of a 4-day low-protein vegetarian diet compared to a mixed diet (0.8 ± 1.11 g/kg BW vs. 1.59 ± 0.28 g/kg BW) in recreationally active men. They observed significantly increased oxygen uptake at different exercise intensities, suggesting that submaximal cycling economy was poorer after a low-protein vegetarian diet [28]. Since the protein intake was restricted and no typical vegetarian diet was studied, the results may not be evident. Moreover, research of Hietavala indicated that a food selection with a high proportion of plant foods may favorably affect the acid-base status and thus potentially positively impact performance [34]. However, a recent review showed no impact on exercise capacity through a diet rich in basic substances [35]. To date, long-term intervention studies are lacking in order to be able to make clear statements about the effect of a vegetarian/vegan diet on exercise capacity.

With increasing intensity of physical activity, an anaerobic energy supply predominates with increasing lactate production. Although vegans had a higher dietary carbohydrate intake in comparison to the other two groups (VEG: 4.66 ± 1.79 vs. OMN: 3.87 ± 1.34 vs. LOV: 3.76 ± 1.55 g/kg BW) no differences regarding submaximal and maximal lactate as well as glucose values were observed between the groups, suggesting no significant influence of diet on glucose utilization. Several factors can affect lactate kinetics during incremental exercise like previous exercise activities, water balance and caffeine consumption [36]. Furthermore, if the intramuscular glycogen stores are emptied, the rate of glycolysis is severely impaired and consequently, lactate production is reduced. It could be suggested that the individual response to exercise training have a stronger impact on exercise capacity than the consumption of meat or animal products, a phenomenon partly attributable to the sex and genetic background of an individual but still incompletely understood [37, 38].

We found a comparable BMI and body composition in all three groups. In contrast, Hanne et al. found a higher body fat mass in female omnivorous athletes compared to vegetarians [25]. However, so far there are no comparative data of vegan athletes. Results of the 24 h dietary recalls did not agree with a study by Lynch et al. [14] since the nutrient intake data of the present LOV and OMN in our study were comparable. Only the VEG group consumed the typically higher amounts of carbohydrates, fiber, magnesium, iron and folate, and less fat and vitamin B_12_.

As a limitation, the present study did not determine oxygen uptake, which would be an interesting parameter assessing the efficiency of cardiorespiratory fitness during physical activity until exhaustion. Further, 24 h dietary recalls may represent not the usual, but the current nutrient intake and have disadvantages regarding rare foods and subjective influences on the stated amounts of consumption. We performed tests on a bicycle as a standardized and save method for assessing exercise capacity and lactate kinetics during exhaustive exercise testings. However, the use of a bicycle instead of a treadmill for runners is a potential limitation of our study.

## Conclusion

Taken into account the aforementioned limitations, the results suggest that there are no differences in exercise capacity between vegan, lacto-ovo-vegetarians and omnivorous recreational runners. Given current data we conclude, that a lacto-ovo-vegetarian and also vegan diet might be suitable alternatives for recreational athletes. Further long-term intervention studies are needed to clarify the influence of a vegetarian and especially vegan diet on an individual’s exercise capacity.

## Abbreviations

[glc]: glucose concentration
[lac]: lactate concentrations
ANOVA: one-way analysis of variance
BCM: body cell mass
BMI: body mass index
BW: body weight
FFM: fat free mass
GXT: graded exercise test
HR: heart rate
LBM: lean body mass
LOV: lacto-ovo-vegetarian athletes
n.s.: not significant
OMN: omnivorous athletes
P_maxBW_: maximum power related to body weight
P_maxLBM_: maximum power output related to lean body mass
SD: standard deviation
TBW: total body water
VEG: vegan athletes
VO_2 max_: maximal oxygen consumption
